# Supplementary material for: Not All Field Margins Are Equally Useful: Effects of the Vegetation Structure of Margins on Cereal Aphids and Their Natural Enemies
Source: Insects. 2023 Feb 3;14(2):156. doi: 10.3390/insects14020156 (PMC9961275; doi:10.3390/insects14020156)
Supplement: Supplementary file 1 [file insects-14-00156-s001.zip › insects-2189560-supplementary.pdf]

Table S1. Absolute frequencies of Hemiptera: Aphididae by sampling area and year.

Species name	Gallecs			Cabrianes (360 t.)	Cardona (520 t.)	L'Espunyola (700 t.)	Moià (680 t.)
	2014 (3290 t.)	2015 (2340 t.)	2016 (3750 t.)				
Anoecia corni (Fabricius, 1775)	1	0	0	0	0	0	0
Diuraphis noxia(Kurdjumov, 1913)	740	137	2	11	22	92	327
Metopolophium dirhodum (Walker, 1849)	8358	478	1797	18	129	792	230
Metopolophium festucae-cerealium Stroyan, 1982	14	23	1	0	0	2	0
Protoaphis sp. Börner, 1952	0	1	0	0	0	0	0
Rhopalosiphum maidis (Fitch, 1856)	1	70	14	0	0	2	0
Rhopalosiphum padi (Linnaeus, 1758)	5091	674	3839	10	2	219	142
Schizaphis graminum (Rondani, 1852)	8153	1553	155	25	16	64	47
Sipha elegans del Guercio, 1905	10,454	1239	829	22	1	15	2
Sipha flava (Forbes, 1884)	0	51	0	0	0	0	0
Sipha maydis Passerini, 1860	1079	186	25	0	0	1	1
Sitobion avenae (Fabricius 1775)	28,399	3258	2656	209	532	806	1061
Sitobion fragariae (Walker, 1848)	0	77	0	0	0	0	29
unknown	168	30	37	1	4	42	29

We also detected some aphid species not related to cereal: *Acyrthosiphon pisum* (Harris, 1776), *Aphis sp*. Linnaeus 1758, *Aploneura lentisci* (Passerini, 1856), *Brachycaudus sp.* van der Goot, 1913, *Phorodon humuli* (Schrank, 1801) and *Protaphis sp*. Börner, 1952. We considered them accidental catches and decided to omit them in subsequent analyses.

Table S2: Absolute frequencies of Hymenoptera: Braconidae: Aphidiinae by sampling area and year. In parentheses, total number of tillers examined.

	**Gallecs**						
**Hymenoptera: Braconidae: Aphidiinae**	**2014 (3290 t.)**	**2015 (2340 t.)**	**2016 (3750 t.)**	**Cabrianes (360 t.)**	**Cardona (520 t.)**	**L'Espunyola (700 t.)**	**Moià (680 t.)**
Adialytus ambiguus (Haliday, 1834)	23	3	2	0	0	0	0
Aphidius ervi Haliday, 1834	47	11	29	1	1	4	1
Aphidius matricariae Haliday, 1834	0	0	1	0	0	0	0
Aphidius rhopalosiphi de Stefani-Pérez, 1902	25	3	10	1	0	1	3
Aphidius sp.	2	1	0	0	0	1	0
Aphidius uzbekistanicus Luzhetzki, 1960	280	40	10	4	1	4	3
Diaeretiella rapae (M'Intosh, 1855)	1	2	0	0	0	0	0
Euaphidius cingulatus (Ruthe, 1859)	1	0	0	0	0	0	0
Lysiphlebus testaceipes (Cresson 1880)	20	0	0	0	0	0	0
Praon volucre (Haliday, 1833)	8	6	5	0	1	9	7
Trioxys sp.	1	0	0	0	0	0	0

Table S3: Absolute frequencies of hyperparasitoids by sampling area and year. In parentheses, total number of tillers examined.

	Gallecs						
	**2014 (3290 t.)**	**2015 (2340 t.)**	**2016 (3750 t.)**	**Cabrianes (360 t.)**	**Cardona (520 t.)**	**L'Espunyola (700 t.)**	**Moià (680 t.)**
**Hymenoptera: Chalcidoidea: Encyrtidae**							
Syrphophagus aphidivorus (Mayr, 1876)	31	9	7	0	0	4	1
**Hymenoptera: Cynipoidea: Figitidae**							
Alloxysta arcuata (Kieffer, 1902)	0	1	0	0	0	0	0
Alloxysta brevis (Thomson, 1862)*.*	3	0	0	0	0	0	0
Alloxysta fracticornis (Thomson, 1862)	0	0	0	0	0	1	0
Alloxysta glebaria Hellen, 1963	0	2	0	0	0	0	0
Alloxysta victrix (Westwood, 1833)	17	7	18	0	0	15	0
Phaenoglyphis villosa (Hartig 1841)	1	0	1	0	0	0	0

(Continued)

**Hymenoptera: Chalcidoidea: Pteromalidae**							
Asaphes suspensus (Nees, 1834)	9	2	0	0	0	12	2
Asaphes vulgaris Walker, 1834	2	0	0	0	0	0	0
Pachyneuron aphidis (Bouché, 1834)	570	51	0	0	0	0	0
Pachyneuron muscarum (Linnaeus, 1758)	1	0	0	0	0	0	0
Pachyneuron solitarium (Hartig, 1838)	7	1	0	0	0	0	0
Pachyneuron sp. Walker, 1833	3	32	0	0	0	0	0
unknown	0	0	34	0	0	0	0
